# Therapeutic impact of epidermal growth factor receptor tyrosine kinase inhibitor with various treatment combinations for advanced lung adenocarcinoma

**DOI:** 10.1186/s12957-023-03203-6

**Published:** 2023-10-13

**Authors:** Ying-Yi Chen, Kuan-Hsun Lin, Yen-Shou Kuo, Yuan-Ming Tsai, Hsu-Kai Huang, Tsai-Wang Huang

**Affiliations:** 1 Division of Thoracic Surgery, Department of Surgery, Tri-Service General Hospital, National Defense Medical Center, Taipei, Republic of China; 2 Graduate Institute of Medical Science, National Defense Medical Center, Taipei, Republic of China

**Keywords:** Epidermal growth factor receptor, Lung adenocarcinoma, Tyrosine kinase inhibitor, Salvage surgery

## Abstract

**Objectives:**

Tyrosine kinase inhibitors (TKIs) are the primary therapeutic option for patients with advanced-stage epidermal growth factor receptor-mutant (EGFR-m) lung adenocarcinoma. However, the role of EGFR-TKIs in advanced-stage lung cancer is uncertain regardless of therapeutic methods. This study investigated the outcome of the impact of epidermal growth factor receptor (EGFR)-TKI in patients with advanced lung adenocarcinoma treated with various therapeutic strategies.

**Methods:**

This retrospective analysis used cancer registry data from 1159 patients with lung cancer treated between January 2015 and December 2017 at Tri-Service General Hospital. Only patients with lung adenocarcinoma stages 3B and four were selected for the study. All lung adenocarcinoma patients with ever TKI treatment had an EGFR mutation.

**Results:**

Three-hundred sixty-two patients with advanced lung adenocarcinoma with complete medical records were enrolled. According to personalized therapeutic processes, they were divided into nine groups: only TKI treatment, only chemotherapy (CT), TKI with lung cancer salvage surgery, TKI with CT, TKI with radiotherapy (RT), CT with lung cancer salvage surgery, CT with RT, TKI with CT, and lung cancer salvage surgery. A multivariate Cox regression analysis showed TKI with lung cancer salvage surgery (HR: 4.675, *p* = 0.005) is the only good prognostic treatment. The poor predictors for overall survival were only CT (HR: 0.336, *p* = 0.048) and TKI with CT (HR: 0.359, *p* = 0.023). Kaplan–Meier survival analysis showed a statistical significance in an average overall survival (OS) of ever TKI treatment and never TKI treatment (33.24 vs. 17.64 months, *p* < 0.001). Furthermore, TKI usage duration was statistically increased in TKI with lung cancer salvage surgery (40.4 ± 20.7 vs 14.96 ± 13.13 months, *p* < 0.001). The survival rate (*p* = 0.033) and OS (*p* < 0.001) in lung cancer salvage surgery were statistically better than the group of TKI without surgery.

**Conclusion:**

The best therapeutic strategy for advanced lung adenocarcinoma is TKI with lung cancer salvage surgery, according to significantly longer OS and better survival. It also prolonged TKI usage. Mutated EGFR lung adenocarcinoma patients with ever TKI treatment had significantly better survival than with other treatments. Regardless of the combination of other treatments, EGFR mutation with TKI therapy is recommended as a positive prognostic factor for patients with lung adenocarcinoma.

**Supplementary Information:**

The online version contains supplementary material available at 10.1186/s12957-023-03203-6.

## Introduction

Non-small cell lung cancer (NSCLC) accounts for approximately 80% of all lung cancers and is the leading cause of cancer deaths worldwide [1]. From the recent meta-analysis [2], the overall epidermal growth factor receptor (EGFR) mutations prevalence in Asian and European regions was estimated to be 49.1% and 12.8%, respectively, and most sub-mutations were actionable: exon 19 deletions (49.2% [Asia]; 48.4% [Europe]) and exon 21 L858R substitutions (41.1% [Asia]; 29.9% [Europe]). A Taiwan cohort study [3] enrolled a total of 1772 patients with lung adenocarcinoma (LUAD) and EGFR mutations were identified in 987 (55.7%). The discovery of oncogenic driver mutations in the EGFR gene was a significant advancement in NSCLC diagnosis and treatment. In the past 10 years, tyrosine kinase inhibitors (TKIs) have been advocated as a treatment for a variety of malignancies [4]. EGFR-TKI suppresses EGFR signaling overactivation and is particularly effective in NSCLC patients with EGFR-activating mutations. EGFR-TKIs have a promising therapeutic response that targets LUAD; however, drug resistance causes the development of progressive disease [5]. Clinical trials [6, 7] show that neoadjuvant EGFR-TKI therapy for locally progressed EGFRm NSCLC is safe and effective. These targeted TKI medicines are showing encouraging outcomes with locally advanced disease [8–10].

EGFR-TKI in patients with EGFR-mutated LUAD had more and more potential benefits with a combination of other therapeutics for all stages based on precision medicine [6, 9, 11–14]. Otherwise, the lung cancer salvage surgery for an advanced lung cancer providing better survival was well established [15–17], and the benefits were proven by the cohort study [18] and clinical trials [6, 9, 14].

However, TKI resistance has been unchangeable until now, and it is also an interesting issue for molecular science. There is no real-world data regarding combination strategies with EGFR-TKI for the overall survival of patients with LUAD. Based on effective TKI treatment results, it is recognized that prolonging TKI usage or preventing TKI resistance can increase the OS of patients with EGFR mutation LUAD. Therefore, this study investigated the outcome of EGFR-TKI in patients with advanced-stage LUAD treated with different therapeutic strategies.

## Materials and methods

We reviewed 1159 cases of patients with NSCLC from the lung cancer registry treated from January 2015 to December 2017 at the Tri-Service General Hospital. All patients were diagnosed pathologically with LUAD. Amplification refractory mutation system-polymerase chain reaction (ARMS-PCR) confirmed the nature of EGFR mutations. All patients’ NSCLC stages were determined by pathological detection and/or standardized uptake value in positron emission tomography/computed tomography (PET/CT). The stage of the primary tumor (T), lymph node (N), and metastasis (M) was determined based on the American Joint Committee on Cancer (AJCC) 8th edition TNM staging for NSCLC [19]. According to personalized therapeutic processes, they were divided into nine groups: only TKI treatment, only chemotherapy (CT), TKI with lung cancer salvage surgery, TKI with CT, TKI with radiotherapy (RT), CT with lung cancer salvage surgery, CT with RT, TKI with CT, and lung cancer salvage surgery. After EGFR-TKI therapy, patients with pre-induction N3 illness experienced downstaging, and their N3 lymph nodes were negative, as validated by PET/CT and ultrasound-guided fine-needle aspiration. Patients undergoing a surgical biopsy for diagnostic purposes were excluded. All patients in the TKI treatment group with lung cancer salvage surgery must undergo complete surgical resection of primary lung cancer with EGFR-TKI cessation for at least 1 week, and all metastases were treated under control or remission before the surgery. One week after surgery, the TKI treatment is ongoing. All the patients were reassessed for resectability. Lung cancer surgical procedures plus systematic lymph node dissection were performed on all patients in the TKI treatment group with the operation. Exclusion criteria were as follows: (1) a history of other cancers within 5 years, (2) primary resistance to EGFR-TKIs, and (3) loss to follow-up. Frequently, the two chemotherapeutic agents are combined to treat lung cancer. If two or more drugs are used together, they are often cisplatin or carboplatin combined with one other drug. Other drug mixtures, like gemcitabine with vinorelbine or paclitaxel, are sometimes used instead.

Propensity score matching with smoking habit, clinical stage, gender, and EGFR mutation genes in patients with LUAD under TKI treatment with and without lung cancer salvage surgery was also determined to further evaluate therapeutic outcomes. This study was approved by the Institutional Review Board/Ethics Committee of Tri-Service General Hospital (C202105025).

### Parameters

Demographic and clinical data, including gender, age, clinical stage, histology, performance status, driver-gene status, the overall duration of targeted therapy, surgical procedure, targeted therapy agents, pathological diagnosis, postoperative therapy, and survival data, were reviewed from the electronic medical records database.

We used biopsy tissue samples (bronchoscopy, endobronchial ultrasound, CT-guided needle biopsy, or navigational bronchoscopy with biopsy) for EGFR mutation tests (Roche cobas® EGFR Mutation Test v2 07248563190 24 tests kit) before TKI treatment.

First-generation (erlotinib and gefitinib) and second-generation TKI agents (afatinib) were used in our study cohort.

### Follow-up

Outpatient visits and phone calls were used for follow-up. Postoperative patients underwent physical examinations and chest CT scans every 3 months in the first year, every 6 months in the next 2 to 5 years, and annually after that. Brain contrast-enhanced magnetic resonance imaging, abdomen ultrasonography, and nuclear bone scans were conducted annually or as determined by clinicians as necessary. Physical exams and chest CTs were performed every 3 months on nonsurgical patients. December 30, 2022, was the cutoff date. Overall survival (OS) was the time between the beginning of treatment and death or the end of the final follow-up. Patients without recurrence or death had their data suppressed at the last follow-up.

### Statistical analysis

Descriptive data were expressed as means ± standard deviations unless otherwise specified. Student’s *t*-test (two-tailed) was used to analyze continuous variables. The chi-square test was used to compare categorical variables between the groups with and without survival status. *p*-values less than 0.05 were statistically significant. All statistical analyses were performed using SPSS version 22.0 (IBM Corp., Armonk, NY, USA).

## Results

**Fig. 1:**
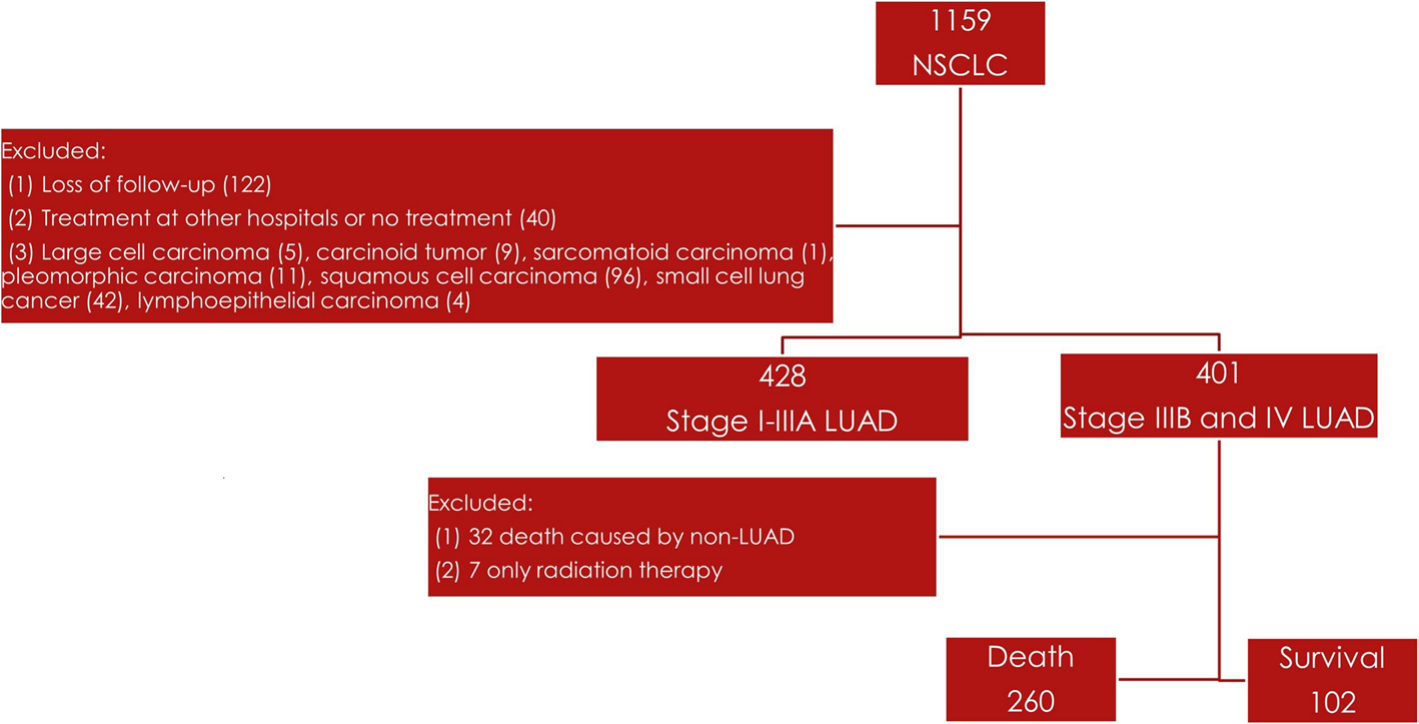
Flowchart of patient selection in our observation study. Total of 362 patients with stages IIIB and IV lung adenocarcinoma were enrolled from lung cancer registry database of 1159 patients with non-small cell lung cancer since 2015/01 to 2017/12

A total of 362 patients with LUAD with clinical stages IIIB and IV were included in this retrospective study (Fig. 1). The patient characteristics are summarized in Table 1. The median age of the survival and death groups was 65.56 ± 12.395 and 66.08 ± 12.081 years (*p* = 0.712). Most patients in the survival group were women (55.88%) and nonsmokers (68.63%). There were 244 (67.4%) patients with EGFR mutation, and all were ever treated with TKI agents. No significant difference was observed in the distribution of gender (*p* = 0.256), smoking history (*p* = 0.055), exon 19 deletion (*p* = 0.453), L858R (*p* = 0.071), exon 18 mutation (*p* = 0.273), and exon 20 insertion (*p* = 0.683) between the survival and death groups. The survival group was significantly predominant in only TKI treatment (*p* = 0.037), TKI with OP (9.8 vs. 2.31%, *p* = 0.002), ever TKI treatment (79.41 vs. 62.69%, *p* = 0.002), clinical stage IIIB (16.67 vs. 8.08%, *p* = 0.001), clinical stage IVA (29.41 vs. 16.92%, *p* = 0.008), and clinical stage IVB (37.27 vs. 25.77%, *p* = 0.047) compared with therapeutic strategies in patients in the death group. However, there were significantly less survival observed in patients in clinical stage IVC (14.71 vs. 49.62%, *p* < 0.001) and more in patients with wild type in the death group (19.61 vs. 35%, *p* = 0.004). There was a significant difference with better OS in the survival group (44.79 ± 16.24 vs. 14.63 ± 11.19 months, *p* < 0.001). The number of metastases in the death group was all significantly more than in survival group (lung or pleura, *p* = 0.002; liver, *p* < 0.001; brain, *p* < 0.001; adrenal gland, *p* = 0.001; bone, *p* < 0.001).

**Table 1:** Patient demographics of lung adenocarcinoma patients with stages IIIB and IV

	Patient demographics of lung adenocarcinoma patients with stages IIIB and IV. Survival (*n* = 102)	Death (*n* = 260)	***P-value***	
Age	65.56 ± 12.395	66.08 ± 12.081	0.712	
Gender				
Male	45 (44.12%)	132 (50.77%)	0.256	
Female	57 (55.88%)	128 (49.23%)		
Smoking				
Yes	32 (31.37%)	110 (42.31%)	0.055	
None	70 (68.63%)	150 (57.69%)		
Clinical stage				
IIIB	17 (16.67%)	21 (8.08%)	**0.001**	
IVA m1a	30 (29.41%)	44 (16.92%)	**0.008**	
IVA m1b	37 (37.27%)	67 (25.77%)	**0.047**	
IVB	15 (14.71%)	129 (49.62%)	**< 0.001**	
Metastases				
Lung or pleura	54 (52.94%)	182 (70%)	**0.002**	
Liver	12 (11.76%)	77 (29.62%)	**< 0.001**	
Brain	4 (3.92%)	90 (34.62%)	**< 0.001**	
Adrenal gland	7 (6.86%)	57 (21.92%)	**0.001**	
Bone	36 (35.29%)	161 (61.92%)	**< 0.001**	
EGFR				
Exon 19 deletion	38 (37.25%)	86 (33.08%)	0.453	
L858R	39 (38.24%)	74 (18.46%)	0.071	
Exon 18 mutation	4 (3.92%)	5 (1.92%)	0.273	
Wild type	20 (19.61%)	91 (35%)	**0.004**	
Exon 20 insertion	1 (0.98%)	4 (1.54%)	0.683	
Treatment				
Only TKI	41 (40.20%)	75 (28.85%)		**0.037**
Only CT	5 (4.90%)	47 (18.08%)		**0.001**
TKI with OP	10 (9.80%)	6 (2.31%)		**0.002**
TKI with CT	9 (8.82%)	45 (17.31%)		**0.042**
TKI with RT	20 (19.61%)	36 (13.85%)		0.174
CT with OP	2 (1.96%)	4 (1.54%)		0.778
CT with RT	14 (13.73%)	44 (16.92%)		0.457
TKI with CT and OP	1 (0.98%)	3 (1.15%)		0.887
Ever TKI	81 (79.41%)	163 (62.69%)		**0.002**
Overall survival	44.79 ± 16.24	14.63 ± 11.19		**< 0.001**

*P*-value < 0.005 is considered as significant difference

*Abbreviations*: *EGFR* Epidermal growth factor receptor, *TKI* Tyrosine kinase inhibitor, *CT* Chemotherapy, *OP* Operation (lung cancer salvage surgery), *RT* Radiation therapy

Prognostic factors for survival in stages IIIB and IV LUAD identified by univariate Cox regression analysis are shown in Table 2. There were significant differences in good prognostic factors in only TKI treatment (*HR*: 1.658, 95% *CI*: 1.028–2.674, *p* = 0.038), TKI treatment with lung cancer salvage surgery (*HR*: 4.601, 95% *CI*: 1.627–13.02, *p* = 0.004), and ever TKI treatment (*HR*: 2.295, 95% *CI*: 1.335–3.946, *p* = 0.003). Male patients (*HR*: 0.776, 95% *CI*: 0.483–1.213, *p* = 0.255), smoking (*HR*: 0.623, 95% *CI*: 0.384–1.013, *p* = 0.056), age (*HR*: 0.996, 95% *CI*: 0.978–1.015, *p* = 0.711), TKI with radiation therapy (*HR*: 1.518, 95% *CI*: 0.831–2.772, *p* = 0.175), chemotherapy with lung cancer salvage surgery (*HR*: 1.28, 95% CI: 0.231–7.099, *p* = 0.778), chemotherapy with radiotherapy (*HR*: 0.781, 95% *CI*: 0.408–1.497, *p* = 0.456), and TKI with chemotherapy and operation (*HR*: 0.848, 95% *CI*: 0.087–8.250, *p* = 0.887) were not statistically significant. Poor prognostic factors with significant differences were only chemotherapy (*HR*: 0.234, 95% *CI*: 0.090–0.606, *p* = 0.003) and TKI treatment with chemotherapy (*HR*: 0.462, 95% *CI*: 0.217–0.985, *p* = 0.045).

**Table 2:** Univariate and multivariate Cox regression analysis to identify prognostic factors for survival in stages IIIB and IV lung adenocarcinoma

	**HR**	**95% *CI***	***P-value***	**HR**	**95% *CI***	***P-value***
Age	0.996	0.978–1.015	0.711			
Gender						
Male vs female	0.776	0.483–1.213	0.255			
Smoking	0.623	0.384–1.013	0.056			
Treatment						
Only TKI	**1.658**	**1.028**–**2.674**	**0.038**	0.972	0.510–1.854	0.932
Only CT	**0.234**	**0.090**–**0.606**	**0.003**	**0.336**	**0.114**–**0.990**	**0.048**
TKI + OP	**4.601**	**1.627**–**13.02**	**0.004**	**4.675**	**1.578**–**13.85**	**0.005**
TKI + CT	**0.462**	**0.217**–**0.985**	**0.045**	**0.359**	**0.149**–**0.867**	**0.023**
TKI + RT	1.518	0.831–2.772	0.175			
CT + OP	1.280	0.231–7.099	0.77			
CT + RT	0.781	0.408–1.497	0.456			
TKI + CT + OP	0.848	0.087–8.250	0.887			
Ever TKI	**2.295**	**1.335**–**3.946**	**0.003**	1.774	0.830–3.791	0.139

*P*-value < 0.005 is considered as significant difference

*Abbreviations*: *HR* Hazard ratio, *CI* Confidence interval, *TKI* Tyrosine kinase inhibitor, *CT* Chemotherapy, *OP* Operation (lung cancer salvage surgery), *RT* Radiation therapy

**Fig. 2:**
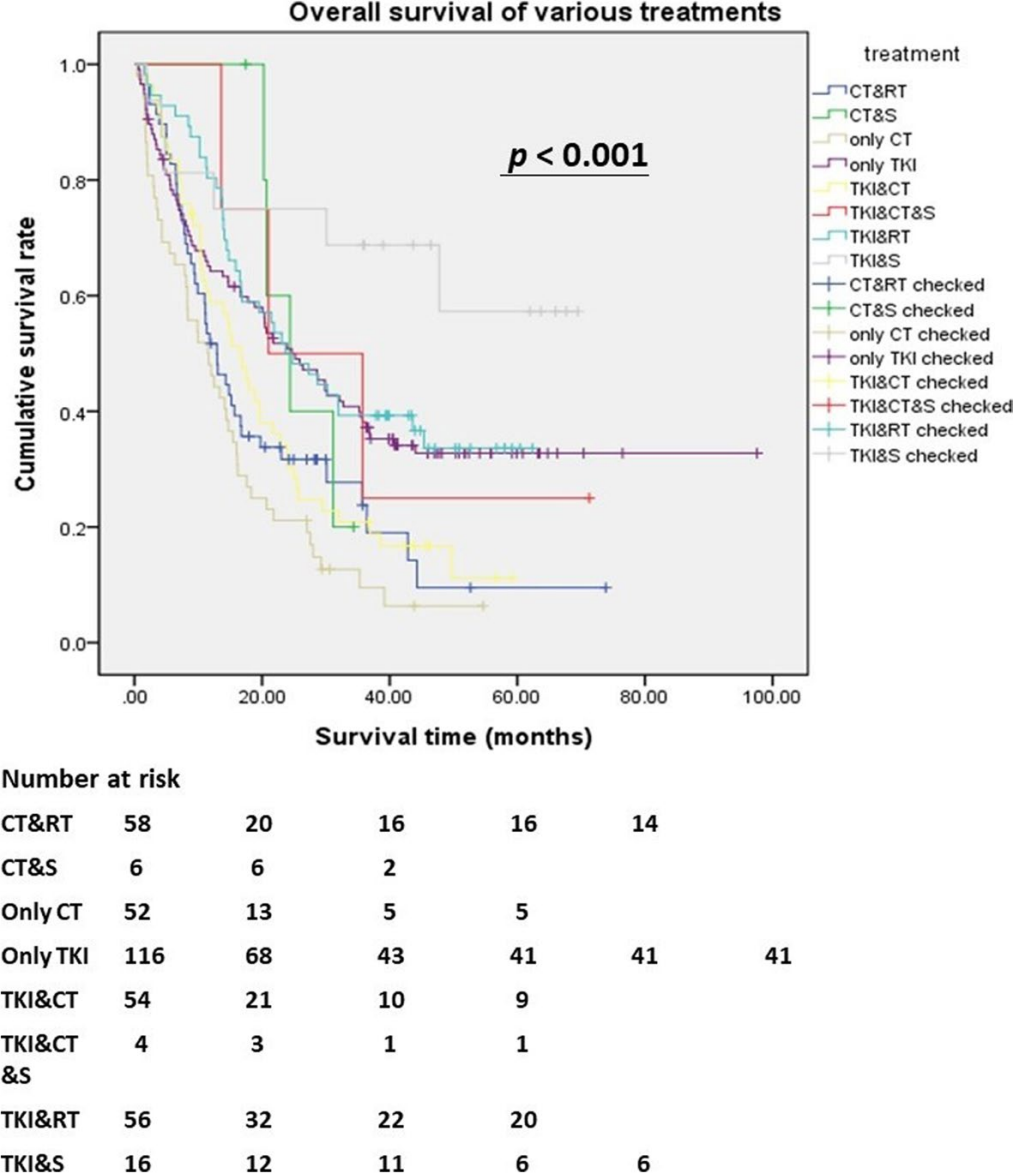
Kaplan–Meier survival curve for various treatments of stages IIIB and IV lung adenocarcinoma. Patients with TKI treatment and lung cancer surgery had the best overall survival compared with other therapies (*p* < 0.001). CT, chemotherapy; RT, radiation therapy; S, surgery; TKI, tyrosine kinase inhibitor

**Fig. 3:**
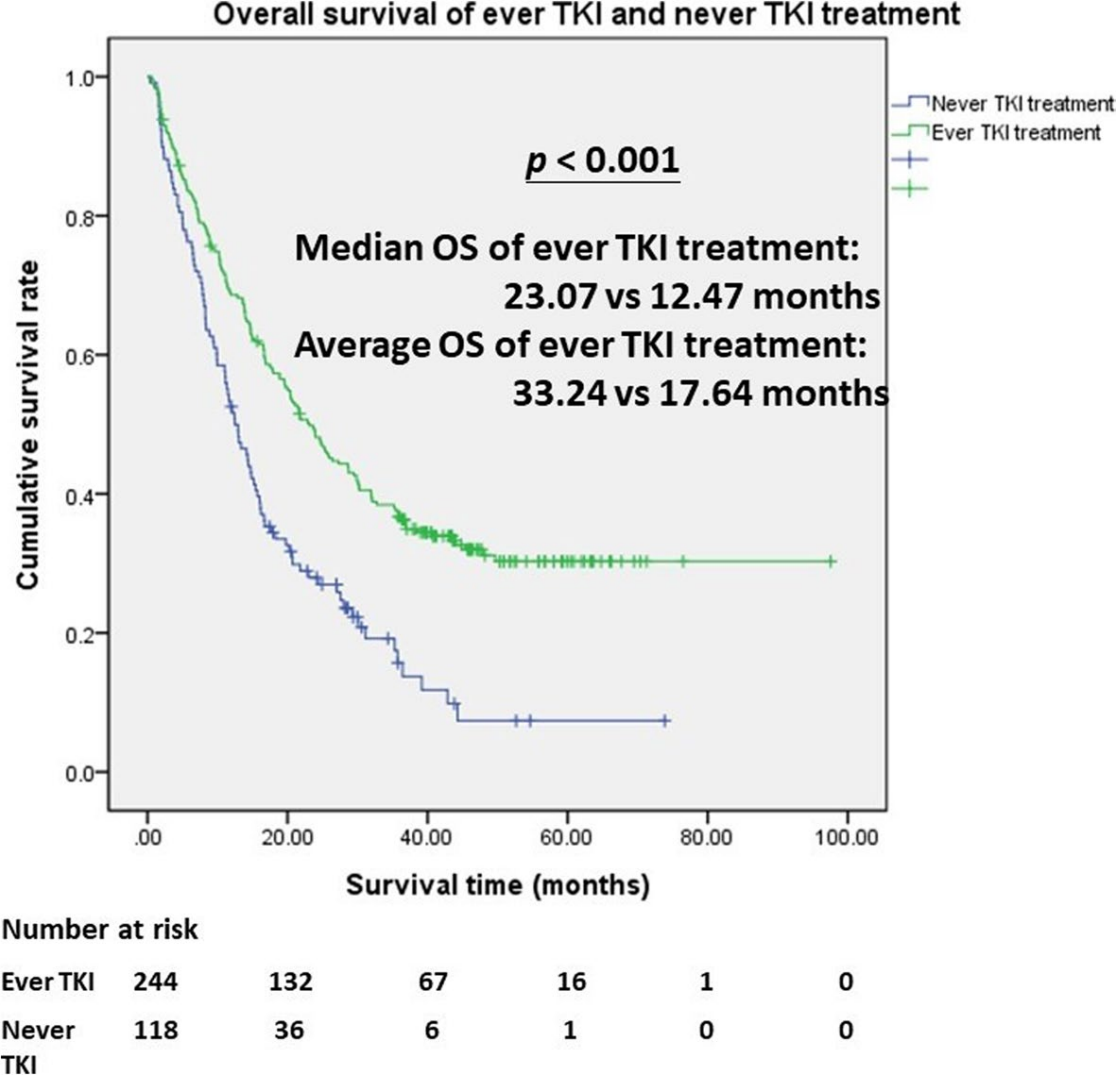
Kaplan–Meier survival curve of comparison with ever versus never TKI treatments in stages IIIB and IV lung adenocarcinoma. Patients with ever TKI treatment got median overall survival 23.07 months and average overall survival 33.24 months, which is better than patients with never TKI treatment (*p* < 0.001)

In the multivariate Cox regression analysis, the independent predictor for better survival was TKI treatment with lung cancer salvage surgery (*HR*: 4.675, 95% *CI*: 1.578–13.85, *p* = 0.005). The independent predictors for poor survival were only chemotherapy (*HR*: 0.336, 95% *CI*: 0.114–0.990, *p* = 0.048) and TKI treatment with chemotherapy (*HR*: 0.426, 95% *CI*: 0.121–0.668, *p* = 0.004). Ever TKI treatment and only TKI treatment were not independent predictors. Figure 2 shows the survival curve of all kinds of treatment. Patients with TKI treatment and lung cancer salvage surgery had the best survival. To further evaluate TKI effects, Fig. 3 shows that patients with ever TKI treatment had significantly better survival than never TKI treatment (*p* < 0.001). The median OS of ever TKI treatment compared with never TKI treatment was 23.07 vs. 12.47 months. The mean OS of ever TKI treatment compared with never TKI treatment was 33.24 vs. 17.64 months.

Table 3 shows the general characteristics of stage IIIB and IV LUAD patients under TKI treatment with and without lung cancer salvage surgery by propensity score matching with control age, gender, smoking habit, EGFR mutation genes, and clinical stage. There were no statistical differences in the lung or pleural metastases (*p* = 0.074), brain metastases (*p* = 0.431), liver metastases (*p* = 1), tumor differentiation (*p* = 0.279), and performance status (*p* = 0.723). Among patients with TKI treatment and lung cancer salvage surgery, 6 patients with lobectomy and 10 patients with sub-lobar resections did not show postoperative complications. There were more patients with metastases of adrenal gland (*p* = 0.014) and bone (*p* = 0.011) in the group of TKI treatment without operation. The TKI treatment group with operation had significant differences in TKI usage duration (40.4 ± 20.7 vs. 14.96 ± 13.13 months, *p* < 0.001), survival status (62.5 vs. 25%, *p* = 0.033), and OS (42.32 ± 19.33 vs. 17.05 ± 13.49 months, *p* < 0.001).

**Table 3:** General characteristics of stages IIIB and IV lung adenocarcinoma patients under TKI treatment with and without lung cancer salvage surgery by propensity score matching with control of age, gender, clinical stage, and smoking habit

	**TKI with OP (*n* = 16)**	**TKI without OP (*n* = 16)**	***P-value***
Age	62.94 + 11.58	63.75 + 10.56	0.837
Gender			
Male	6 (37.5%)	7 (43.75%)	0.729
Female	10 (62.5%)	9 (56.25%)	
Smoking			
Yes	5 (31.25%)	9 (56.25%)	0.164
None	11 (68.75%)	7 (43.75%)	
Grade of differentiation			
Moderate	12 (75%)	9 (56.25%)	0.279
Poor	4 (25%)	7 (43.75%)	
Operation			
Lobectomy	6 (37.5%)		
Wedge or segmentectomy	10 (62.5%)		
Performance status			
0	9 (56.25%)	8 (50%)	0.723
1	7 (43.75%)	8 (50%)	
Clinical stage			
IIIB	1 (6.25%)	0	0.325
IVA m1a	6 (37.5%)	3 (18.75%)	0.252
IVA m1b	2 (12.5%)	6 (37.5%)	0.109
IVB	7 (43.75%)	7 (43.75%)	1
Metastases			
Lung or pleura	15 (93.75%)	11 (68.75%)	0.074
Brain	5 (31.25%)	3 (18.75%)	0.431
Adrenal gland	0	5 (31.25%)	**0.014**
Bone	6 (37.5%)	13 (81.25%)	**0.011**
Liver	6 (37.5%)	6 (37.5%)	1
EGFR mutation			
L858R	6 (37.5%)	6 (37.5%)	1
Exon 19 deletion	10 (62.5%)	10 (62.5%)	
TKI usage duration (month)	**40.4 ± 20.7**	**14.96 ± 13.13**	**< 0.001**
Survival			
Yes	**10 (62.5%)**	**4 (25%)**	**0.033**
None	**6 (37.5%)**	**12 (75%)**	
Overall survival	**42.32 ± 19.33**	**17.05 ± 13.49**	**< 0.001**

*P*-value < 0.005 is considered as significant difference

*Abbreviations*: *TKI* Tyrosine kinase inhibitor, *CT* Chemotherapy, *OP* Operation (lung cancer salvage surgery), *EGFR* Epidermal growth factor receptor

**Fig. 4:**
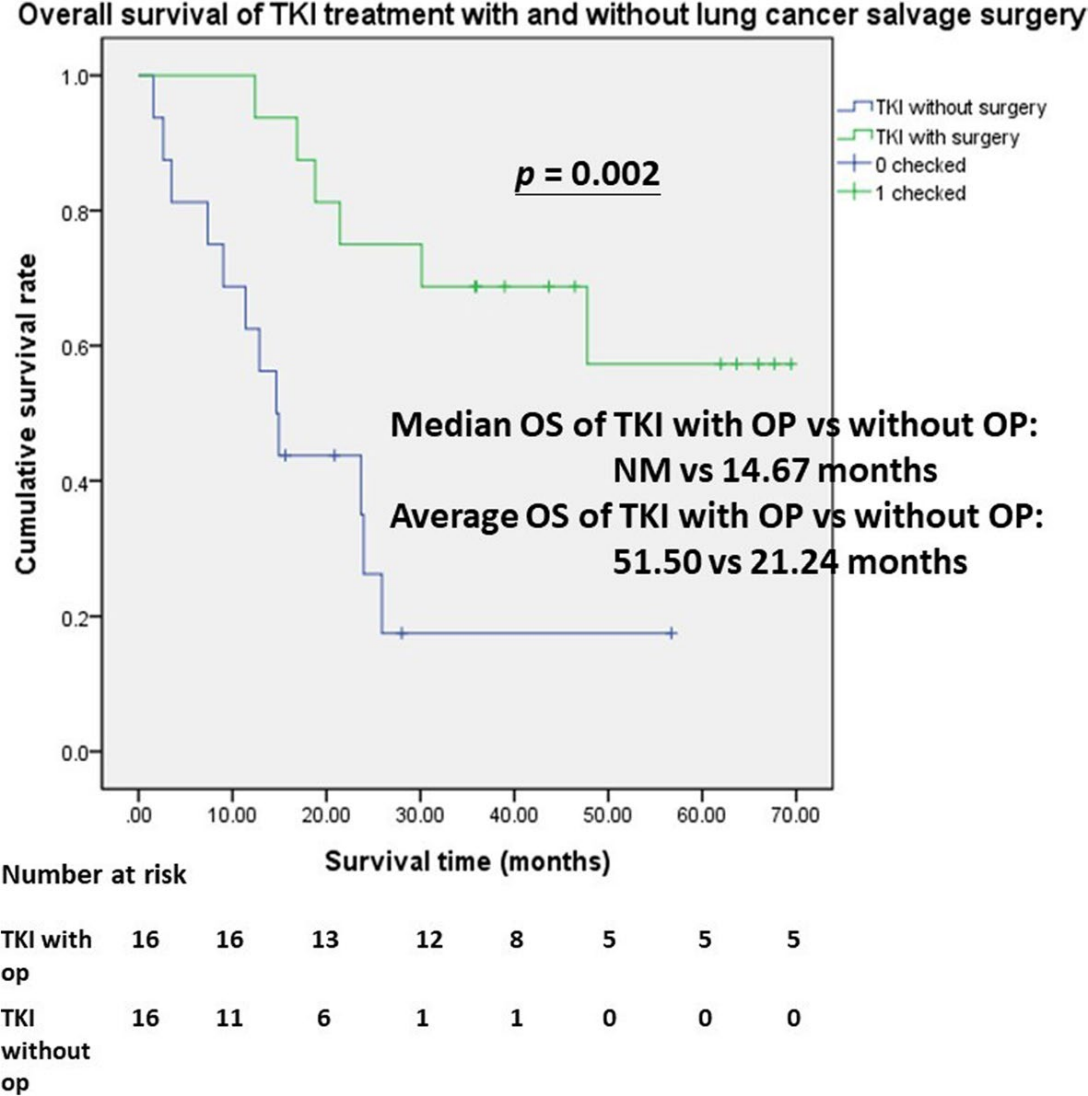
Kaplan–Meier survival curve of TKI treatments with versus without lung cancer surgery in stages IIIB and IV lung adenocarcinoma. Lung adenocarcinoma patients with TKI treatment and lung cancer surgery got a statistically significant better survival (*p* = 0.002) than TKI treatment without operation by propensity score matching. The median overall survival was still not met, and the average overall survival was 51.5 months

Figure 4 shows LUAD patients with TKI treatment, and lung cancer salvage surgery got a statistically significant better survival (*p* = 0.002) than TKI treatment without operation by propensity score matching. The median OS was not yet achieved. The average OS was 51.5 vs. 21.24 months.

## Discussion

After effective targeted therapy, salvage surgery allows complete access to the benefits of surgery and targeted therapies. This is an example of multidisciplinary customized treatment [19]. To address this issue and improve clinical outcomes, numerous additional therapies (primary lung cancer resection, radiation therapy, and chemotherapy) designed for use combined with EGFR-TKIs are being developed. In this study cohort, we retrospectively analyzed the efficiency of combination therapies in patients with advanced lung cancer after targeted therapy. Although the therapeutic role of target therapy with TKI agents in LUAD is well established with better OS and progression-free survival, the destiny of TKI resistance is still unchangeable and a hot issue for molecular biology investigation.

Therefore, we tried to determine the outcomes of combination therapies or not with TKI treatment for patients with advanced LUAD. To our knowledge, this study is the first on this subject, according to real-world data. We used the lung cancer registry data of the Tri-Service General Hospital and found that TKI treatment with lung cancer salvage surgery was a significant prognostic factor for better OS (*HR*: 4.675, *p* = 0.005). Furthermore, lung cancer resection as post-TKI treatment can prolong TKI usage duration (40.4 ± 20.7 vs. 14.96 ± 13.13 months, *p* < 0.001) as well as OS (42.32 ± 19.33 vs. 17.05 ± 13.49 months, *p* < 0.001). This result proved our hypothesis, and primary tumor resection is the best prognostic combination therapy with TKI treatment. According to our experience, it is essential to know about the histologic changes in surgically removed lung cancer specimens following TKI therapy. Only two of sixteen patients switched to T790M after second-generation TKI treatment and shifting the regimen to osimertinib for subsequent targeted therapy. The results of therapeutic outcomes still need large prospective studies or clinical trials to strengthen the evidence.

This activating mutation, seen in up to 50% of NSCLC patients, results in ligand-independent downstream signaling of EGFR, encouraging enhanced malignant cell survival, proliferation, invasion, and metastasis [20]. Fifteen percent of patients with NSCLC had EGFR somatic activating mutations (10–50% depending on demography, with greater frequency in East Asian patients) [21]. Several clinically authorized EGFR-TKIs have response rates of 50–80% [22, 23]. This explains the great success of TKI treatment in our patients by prevalent trend. Due to the emergence of small subpopulations of resistant or tolerant cells, most EGFR-mutant NSCLCs that initially respond to EGFR-TKIs eventually become resistant [24–26]. Several acquire resistance mechanisms which have been identified, including EGFR exon20 T790M mutation, bypass pathway activation, and histologic transformation. A greater comprehension of the underlying causes of drug tolerance and the development of resistance should aid in enhancing the efficacy of cancer treatments [27]. More and more combination medicines were produced and examined for overcoming TKI resistance [13]. However, combination therapy with TKI agents might increase side effects and further resistance with no end to a vicious cycle. Therefore, primary lung cancer resection is suggested because of the huge tumor burden with various genetic backgrounds after TKI treatment with stable or regression conditions.

Few studies have documented the clinical outcomes of salvage surgery following TKI therapy in patients with initially inoperable advanced NSCLC [12, 18, 28, 29]. Ohtaki and colleagues presented a retrospective analysis (a total of 36 patients) based on Japanese national data that demonstrated that salvage surgery after TKI therapy is safe and feasible and may lead to a longer overall survival time (3-year OS after surgery, 75.1%) [12]. However, this study did not compare with the outcome of other therapies and showed us single-arm data. The other three studies were also from the Taiwan Medical Center. Corresponding author Professor Tseng and colleagues from Tainan City of Taiwan presented 29 patients with EGFR-mutant advanced LUAD who underwent salvage surgery after EGFR-TKI treatment [29]. They concluded that the median progression-free survival (PFS) after surgery was 36.4 months, and the median OS was not reached. Following EGFR-TKI therapy for advanced LUAD, salvage surgery is a safe procedure with acceptable intra- and postoperative outcomes. Dr. J. S. Tseng and colleagues from Taichung City of Taiwan showed a retrospective study of 76 patients from a total of 466 with primary tumor resection after TKI treatment who experienced significantly longer progression-free survival (25.1 [95% *CI* 16.6–33.7] vs. 9.4 [95% *CI* 8.4–10.4] months; *HR* 0.40 [95% *CI* 0.30–0.54], *p* < 0.001) and OS (56.8 [95% *CI* 36.3–77.2] vs. 31.8 [95% *CI* 28.2–35.4] months; *HR* 0.57 [95% *CI* 0.39–0.84], *p* = 0.004) [28]. Professor Lin and colleagues presented 29 advanced LUAD patients who underwent EGFR-TKI therapy followed by salvage operation, and the 3-year overall survival was 75.9% [18]. Prospective multicenter investigations are required to validate the outcomes of these prior retrospective studies.

Our study had limitations.

(I) This study was retrospective; therefore, selection bias was inevitable and could have affected the findings. For confirmation, randomized controlled clinical trials are required.
(II) The sample size was small, as radical surgery was not the standard treatment for these patients according to clinical guidelines.
(III) During follow-up, some patients lacked information on their survival status and therapeutic regimens after disease progression or recurrence.

These patients were excluded. Extending the duration of follow-up and analyzing post-recurrence treatment strategies would be the focus of future research.

### Supplementary Information

**Additional file 1.** Surgical patients with T and N stage.

## Data Availability

The data that support the findings of this study are available on request from the corresponding author. The data are not publicly available due to privacy or ethical restrictions.
